# Efavirenz-Based Regimens in Antiretroviral-Naive HIV-Infected Patients: A Systematic Review and Meta-Analysis of Randomized Controlled Trials

**DOI:** 10.1371/journal.pone.0124279

**Published:** 2015-05-01

**Authors:** Joanna Kryst, Paweł Kawalec, Andrzej Pilc

**Affiliations:** 1 Drug Management Department, Institute of Public Health, Faculty of Health Sciences, Jagiellonian University, Krakow, Poland; 2 Department of Neurobiology, Institute of Pharmacology, Polish Academy of Sciences, Krakow, Poland; 3 Independent EBM expert, Kraków, Poland; University of Pittsburgh, UNITED STATES

## Abstract

Efavirenz, a non-nucleoside reverse-transcriptase inhibitor (NNRTI) is one of the most commonly prescribed antiretroviral drugs. The present article provides a systematic overview and meta-analysis of clinical trials comparing efavirenz and other active drugs currently recommended for treatment of HIV-infected, antiretroviral-naive patients. Electronic databases (Pubmed, Embase, the Cochrane Library, Trip Database) were searched up till 23 December 2013 for randomized controlled clinical trials published as a peer-reviewed papers, and concerning efavirenz-based regimens used as initial treatment for HIV infection. Thirty-four studies were included in the systematic review, while twenty-six trials were suitable for the meta-analysis. Efavirenz was compared with drugs from four different classes: NNRTIs other than efavirenz (nevirapine or rilpivirine), integrase strand transfer inhibitors (InSTIs), ritonavir-boosted protease inhibitors (bPI) and chemokine (C-C motif) receptor 5 (CCR5) antagonists (maraviroc), all of them were added to the background regimen. Results of the current meta-analysis showed that efavirenz-based regimens were equally effective as other recommended regimens based on NNRTI, ritonavir-boosted PI or CCR5 antagonist in terms of efficacy outcomes (disease progression and/or death, plasma viral HIV RNA <50 copies/ml) while statistically significant more patients treated with InSTI achieved plasma viral load <50 copies/ml at week 48. In comparison with both InSTI-based and CCR5-based therapy, efavirenz-based treatment was associated with a higher risk of therapy discontinuation due to adverse events. However, comparisons of efevirenz-based treatment with InSTI-based and CCR5-based therapy were based on a limited number of trials, therefore, conclusions from these two comparisons must be confirmed in further reliable randomized controlled studies. Results of our meta-analysis support the present clinical guidelines for antiretroviral-naive, HIV-infected patients, in which efavirenz is one of the most preferred regimens in the analyzed population. Beneficial safety profile of InSTI-based and CCR5-based therapy over efavirenz-based treatment needs further studies.

## Introduction

Highly-active antiretroviral therapy (HAART) with three or more antiretroviral drugs is nowadays a “gold standard” of HIV treatment. HAART has been shown to reduce morbidity and mortality in HIV-infected patients [1–2]. Results from recent studies show that about 80% of treatment-naive patients reached plasma HIV RNA level below detection limit after 48 weeks of HAART therapy (when intent-to-treat (ITT) approach was applied) [3–4]. Currently investigated treatment options concerning new classes of drugs, such as for example chemokine (C-C motif) receptor 5 (CCR5) antagonists and integrase inhibitors (InSTI) may improve efficacy outcomes in HIV-infected patients. Efavirenz belongs to the class of non-nucleoside reverse-transcriptase inhibitors (NNRTIs) and is one of the most commonly prescribed antiretroviral medications in the world [5]. The efficacy and safety of efavirenz were assessed in numerous head-to-head randomized controlled trials (RCTs). Its effectiveness in antiretroviral-naive and treatment-exposed HIV-infected patients was compared with various regimens (mostly PI-based), however there is still a lack of comprehensive review regarding comparison of efavirenz-based therapy with other, actually recommended regimens. Recent practice guidelines of initial treatment in HIV-infected patients, among preferred combinations of antiretroviral drugs mentioned two nucleoside reverse transcriptase inhibitors (NRTIs) plus either a non-nucleoside reverse-transcriptase inhibitor (NNRTI), ritonavir-boosted protease inhibitor (ritonavir-boosted PI) or integrase strand transfer inhibitor (InSTI) [6–8]. In some circumstances, a CCR5 antagonist in combination with two NRTIs are also recommended [6–7]. In the light of numerous trials regarding the use of efavirenz in HIV-infected, antiretroviral-naive patients, we performed systematic review and meta-analysis of randomized controlled trials in order to establish differences between efavirenz-based regimens and other regimens recommended by clinical experts to be used in HIV-infected patients previously untreated with antiretroviral therapy.

## Methods

This review was performed in accordance with the preferred reporting items for systematic reviews and meta-analyses (PRISMA) guidelines [9] and methods described in the Cochrane Handbook [10]. A systematic search of electronic databases and reference lists of all eligible studies published up till 23 December 2013 was conducted in order to identify all relevant studies. The search was conducted in the following databases: Medline via PubMed, EMBASE, the Cochrane Central Register of Controlled Trials (CENTRAL), and the Trip Database. The search strategy included MeSH and EMTREE terms combined the with boolean logical operators AND and OR (Table 1). The search results were restricted to clinical studies and methodological filters were used for the selection of randomized controlled trials (RCTs). No limits were applied for language of articles. The Cochrane Database of Systematic Reviews, PubMed and EMBASE databases were also searched for review articles. We included all randomized controlled trials published as a full text comparing efavirenz with any other, commonly used treatment schedule in adult HIV-infected patients without prior exposure to antiretroviral therapy (studies assessing placebo as a comparator were excluded). Data presented only at conference meetings in abstract form were not included in the systematic review and meta-analysis, as the reliability of such results is lower than published peer-reviewed references. We also excluded studies where efavirenz was admini\\stered to patients in every treatment arm, trials conducted only on children and infants and carried out in HIV-infected patients with other concurrent infectious illnesses, like hepatitis B, hepatitis C or tuberculosis. The following outcomes were assessed: (i) progression of disease or death, (ii) virological response to treatment, and (iii) safety profile (defined as risk of adverse events and discontinuation of the treatment due to adverse events).

**Table 1:** MeSH subject headings and EMTREE keywords used in search strategy construction (last updated: 23.12.2013).

Keywords (combined with boolean logical operators: AND, OR)	
Medical condition	(Viruses, Human Immunodeficiency) OR (AIDS Virus) OR (AIDS Viruses) OR (Virus, AIDS) OR (Viruses, AIDS) OR (HTLV-III) OR (Human Immunodeficiency Virus) OR (Human Immunodeficiency Viruses) OR (Human T Cell Lymphotropic Virus Type III) OR (Human T Lymphotropic Virus Type III) OR (Human T-Cell Leukemia Virus Type III) OR (Human T-Cell Leukemia Virus Type III) OR (Human T-Cell Lymphotropic Virus Type III) OR (Human T-Cell Lymphotropic Virus Type III) OR (Immunodeficiency Virus, Human) OR (Immunodeficiency Viruses, Human) OR (LAV-HTLV-III) OR (Lymphadenopathy-Associated Virus) OR (Lymphadenopathy-Associated Virus) OR (Lymphadenopathy-Associated Viruses) OR (Virus, Lymphadenopathy-Associated) OR (Viruses, Lymphadenopathy-Associated) OR (Virus, Human Immunodeficiency) OR (Acquired Immune Deficiency Syndrome Virus) OR (Acquired Immunodeficiency Syndrome Virus) OR (aids associated lentivirus) OR (aids associated retrovirus) OR (aids associated virus) OR (aids related virus) OR HIV OR (immunodeficiency associated virus) OR (immunodeficiency viruses primate) OR lav OR (LAV (AIDS)) OR (lentiviruses, primate) OR (lymphadenopathy associated retrovirus) OR (Lymphadenopathy associated virus) OR (virus, lymphadenopathy associated)
Intervention	Efavirenz OR EFV OR EFZ OR (efavirenz, (R)-isomer) OR Sustiva OR (L 743726) OR (L-743,726) OR (L-743726) OR (L 743,726) OR Stocrin OR (Merck Sharp and Dohme brand of efavirenz) OR (DMP 266) OR (DMP-266) OR (dmp266) OR efavir OR filginase OR l743726 OR (efavirenz, (S)-isomer) OR (virorrever)
Methodological limits	PubMed: Humans, Randomized Controlled Trial; EMBASE: Humans, Randomized Controlled Trial, Embase only; CENTRAL: No limits applied; word variations have been searched

Two reviewers (J.K., P.K.) performed an independent search and selection process. Disagreements were resolved by discussion, consensus, and arbitration by the third author (A.P.). Full texts of articles were reviewed according to the predefined inclusion or exclusion criteria. The second reviewer (P.K.) verified data extracted by the first author (J.K.). The following data were extracted: study design, characteristics of study participants, interventions, duration of treatment and clinical outcomes. We used Jadad scale [11] (which evaluates studies based on their description of randomization, blinding, and dropouts) to assess the methodological quality of the included studies (Table 2).

**Table 2:** Methodological quality of included RCTs.

Study (acronym if stated)	Jadad score						Allocation concealment
	1	2	3	4	5	Total	
[15] Cohen 2011, THRIVE	1	0	1	1	1	4	Not reported
[16] Gaytán 2004	1	0	0	0	0	1	Not reported
[17] Gazzard 2011, [18] Nelson 2011, SENSE	1	0	1	1	1	4	Not reported
[19] Molina 2011, ECHO	1	0	1	1	1	4	Not reported
[20] Nunez 2002, SENC	1	0	0	0	1	2	Not reported
[21] Pozniak 2010, TMC278-C204	1	0	0	0	1	2	Not reported
[22] van den Berg-Wolf 2008, substudy of FIRST [23]	1	0	0	0	0	1	Not reported
[24] van Leth 2004, 2NN	1	0	0	0	1	2	Described
[25] Vernazza 2013, A5271015	1	0	1	0	1	3	Not reported
[26] Wester 2010, TSHEPO	1	0	0	0	1	2	Not reported
[28] Cohen 2011, GS-236-014	1	0	1	1	1	4	Not reported
[29] Lennox 2009, [30] Lennox 2010, STARTMRK	1	0	1	1	1	4	Described
[31] Markowitz 2007, [32] Markowitz 2009, Protocol 004	1	0	1	1	1	4	Not reported
[33] Sax 2012, [34] Zolopa 2013, GS-US-236-0102	1	0	1	1	1	4	Described
[35] van Lunzen 2012, [36] Stellbrink 2013,SPRING-1	1	0	0	0	1	2	Not reported
[37] Walmsley 2013, SINGLE	1	0	1	1	1	4	Not reported
[38] Albini 2012	1	0	0	0	1	2	Not reported
[39] Bartlett 2006, CLASS	1	0	0	0	1	2	Not reported
[40] Cameron 2008, M03-613	1	0	0	0	1	2	Not reported
[41] Daar 2011, A5202	1	0	0	0	1	2	Described
[42] Echeverría 2010, LAKE	1	0	0	0	1	2	Not reported
[43] Honda 2011	1	0	0	0	1	2	Not reported
[44] Josephson 2010, [45] Edén 2010, [46] Andersson 2013, NORTHIV	1	0	0	0	1	2	Not reported
[47] Kumar 2013, SUPPORT	1	0	0	0	1	2	Not reported
[48] Mallolas 2008, TRIZEFAL	1	0	0	0	1	2	Not reported
[49] Miró 2010, ADVANZ	1	0	0	0	1	2	Not reported
[50] Puls 2010, ALTAIR	1	0	0	0	1	2	Not reported
[51] Ratsela 2010, PHISIDA II	1	0	0	0	1	2	Not reported
[52] Riddler 2008, A5142	1	0	0	0	1	2	Not reported
[53] Sierra-Madero 2010	1	0	0	0	1	2	Not reported
[54] Torti 2005,[55] Torti 2008	1	0	0	0	1	2	Not reported
[56] Copper 2010, [57] Sierra-Madero 2010, MERIT	1	0	1	0	1	3	Not reported
[58] Currier 2008, ASCENT	1	0	0	0	1	2	Not reported
[59] Landovitz 2008	1	0	1	0	1	3	Not reported

**1**—Was the study described as randomized?

**2**—Was the method of randomization described and appropriate?

**3**—Was the study described as double blind?

**4**—Was the method of blinding described and appropriate?

**5**—Were withdrawals and dropouts described?

Reduction of Risk Ratio (RR) was obtained for data showing the benefit of treatment, while for negative endpoints, the increase in RR was assessed, all with 95% confidence intervals (CI). The results obtained from separate trials were combined using appropriate meta-analysis methods. If possible, data from intention-to-treat (ITT) analyses, which assessed patients according to their assigned treatment group were extracted. An inverse variance and the Mantel-Haenszel or DerSimonian-Laird methods were used in dependence on the data input and heterogeneity of test results. The clinical heterogeneity was assessed by examining the characteristics of the studies, whereas the statistical heterogeneity was assessed using the Chi-square test, with a significance level p<0.10. A fixed effects model was used when no statistical heterogeneity was detected, otherwise the random effects model was used. Meta-analysis was performed with RevMan V 5.2 software.

## Results

**Fig 1:**
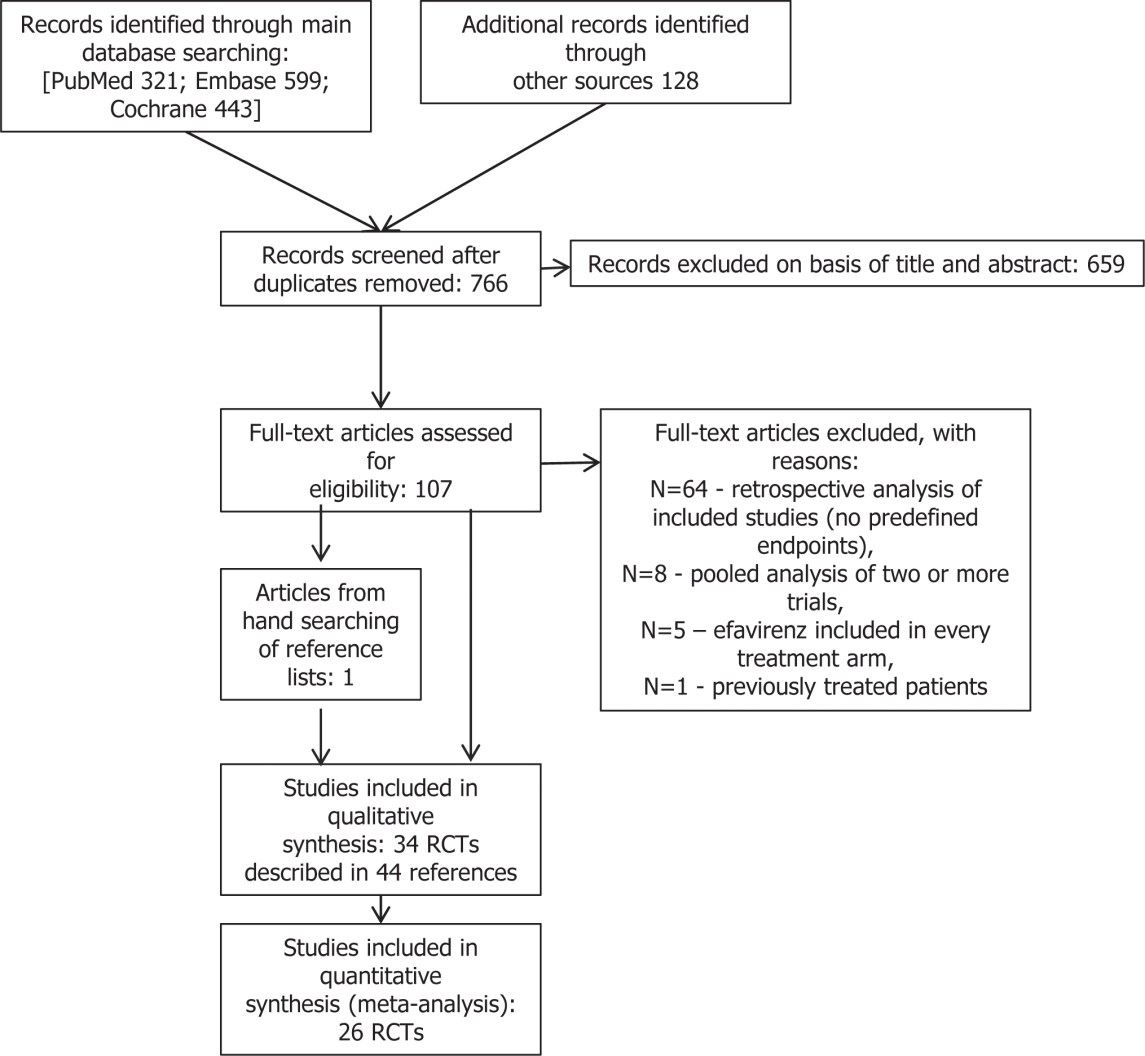
PRISMA flow diagram for selection of studies identified in the systematic review.

The electronic searches yielded 766 items after duplicates were removed. The selection of titles and abstracts resulted in 107 potentially relevant articles, of which 64 references were excluded due to the reasons presented in Fig 1. Finally 34 studies described in 44 references met the predefined inclusion criteria. Twenty-six RCTs were suitable for quantitative synthesis (meta-analysis). The flow of information through the different phases of the systematic review is shown in Fig 1.

Thirty-four randomized controlled trials published in English as peer-reviewed articles were included. These included studies were grouped in the following way. Firstly, background regimen, common in compared groups, was identified. Active drugs added to the common background regimen were considered as comparators. Two different regimens adequate for comparisons with efavirenz were identified: NNRTI (non-nucleoside reverse transcriptase inhibitor), InSTI (integrase strand transfer inhibitor), bPI (ritonavir-boosted protease inhibitor), and CCR5 (CC chemokine receptor type 5), all added to the specified background regimens. We did not take into consideration regimens containing unboosted PI and triple NRTIs regimens as they are no longer used in the clinical practice.

The predefined inclusion criteria for studies included the absence of any prior treatment with antiretroviral therapy, however finally, trials recruiting patients with limited previous exposure to antiretroviral therapy were included (Table 3). Both patient-important endpoints (AIDS disease progression and/or death) and surrogate endpoints (virological response measured with plasma HIV RNA level, pVL) were extracted from the studies. Plasma viral load (pVL) is a globally accepted endpoint used to measure the efficacy of antiretroviral drugs [12]. In the included studies, the virological response was defined as plasma HIV RNA level below 400 and below 50 copies/ml. We assessed only the pVL<50 copies/ml, as the suppression of pVL to 50 copies/ml is a better predictor for durable virological success than a suppression to <400–500 copies/ml [13–14]. For safety analysis an overall risk of grade 3/4 adverse events was assessed when it was possible; otherwise clinical adverse events data were included. We also evaluated the risk of discontinuation of assigned treatment due to treatment toxicity.

**Table 3:** Characteristics of the randomized controlled trials for efavirenz compared to different regimens used to treat antiretroviral–naive HIV-infected adult patients.

Study author, year of publication, study type, sites	Population	Study duration	Interventions*
background regimen (2 NRTIs/2 NRTIs+1 PI) + efavirenz vs background regimen + NNRTI			
[15] Cohen 2011, THRIVE, RCT, double-blind, double-dummy, 98 centers in 21 countries (United States and Puerto Rico, Canada, Australia, Europe, South Africa, Asia, Latin America)	ART-naive, age ≥18 years, pVL >5 000 copies/ml	96 weeks	A: 2 NRTI + EFV, N = 340; B: 2 NRTI + RPV, N = 340; (2 NRTI included: TDF + FTC or AZT + 3TC or ABC + 3TC)
[16] Gaytán 2004, RCT, open-label, 1 center in Mexico	ART-naive, age ≥18 years, pVL >55 000 copies/ml	48 weeks	A: AZT + 3TC + EFV, N = 30; B: AZT + 3TC + NVP, N = 28
[17] Gazzard 2011, [18] Nelson 2011, SENSE, RCT, double-blind, centers in Europe, Russia and Israel	ART-naive, pVL>5 000 copies/ml	48 weeks	A: 2 NRTI + EFV, N = 78; B: 2 NRTI + ETV, N = 79; (2 NRTI included: TDF + FTC or AZT + 3TC or ABC + 3TC)
[19] Molina 2011, ECHO, RCT, double-blind, double-dummy, 112 centers in 21 countries (United States, Canada, Australia, South Africa, Europe, Asia, Latin America)	ART-naive, age ≥18 years, pVL>5 000 copies/ml	96 weeks	A: TDF + FTC + EFV, N = 348; B: TDF + FTC + RPV, N = 346
[20] Núñez 2002, SENC, RCT, open-label, 1 center in Spain	ART-naive, age > 18 years, pVL: 500–100 000 copies/ml	48 weeks	A: ddI + d4T + EFV, N = 31; B: ddI + d4T + NVP, N = 36
[21] Pozniak 2010, TMC278-C204, RCT, open-label, 54 centers in 14 countries (Asia, South Africa, Uganda, Europe, United States, Russia, Puerto Rico, Latin America)	ART-naive, age ≥18 years (≤2 weeks treatment with NRTI and/or PI was allowed), pVL >5 000 copies/ml	96 weeks	A: 2 NRTI + EFV, N = 89; B: 2 NRTI + RPV, N = 279 (25 mg N = 93, 75 mg N = 95, 150 mg N = 91); (2 NRTI included: TDF + FTC or AZT + 3TC)
[22] van den Berg-Wolf 2008, NNRTI substudy of FIRST trial [23], RCT, open-label, 17 clinical trials units at 80 sites in the United States	ART-naive (less than 4 weeks of prior NRTI use or 1 week of 3TC use was allowed), age ≥13 years	median—5 years	patients randomized to NNRTI+NRTIs strategy (N = 110) or PI+NNRTI+NRTIs strategy (118) and then randomized to: A: EFV, N = 111; B: NVP, N = 117 (NRTIs included: ABC + 3TC or ddI + d4T or AZT + 3TC or d4T + 3TC; PI included: NFV, INV, /r
[24] van Leth 2004, 2NN, RCT, open-label, centers in North and South America, Australia, Europe, South Africa and Thailand	ART-naive, age ≥16 years, pVL >5 000 copies/ml	48 weeks	A: d4T+ 3TC+ EFV, N = 400; B: d4T + 3TC + NVP (400 mg once daily), N = 220; C: d4T + 3TC + NVP (200 mg twice daily), N = 387
[25] Vernazza 2013, A5271015, RCT, double-blind, centers in Argentina, Australia, Canada, Italy, Mexico, Poland, South Africa, Switzerland and the United Kingdom	ART-naive (less than 14 days of prior ART was allowed), age ≥18 years, pVL ≥1 000 copies/ml	96 weeks	A: TDF + FTC + EFV, N = 63; B: TDF + FTC + LRV (500 mg), N = 66; C: TDF + FTC + LRV (750 mg), N = 66
[26] Wester 2010, TSHEPO, RCT, open-label, one center in Botswana	ART-naive, age ≥ 18 years, pVL >55 000 copies/ml	3 years	A: 2 NRTI + EFV, N = 325; B: 2 NRTI + NVP, N = 325; (2 NRTI included: AZT + 3TC or AZT + ddI or d4T + 3TC)
background regimen (2 NRTIs) + efavirenz vs background regimen + InSTI			
[28] Cohen 2011, GS-236-014, RCT, double-blinded, double-dummy, mulit-center (centers not described)	ART-naive, age ≥18 years, pVL ≥5 000 copies/ml	48 weeks	A: FTC + TDF + EFV, N = 23; B: FTC + TDF + EVG + COBI, N = 48
[29] Lennox 2009, [30] Lennox 2010, STARTMRK, RCT, double-blind, 67 study centers in Australia, Brazil, Canada, Chile, Colombia, France, Germany, India, Italy, Mexico, Peru, Spain, Thailand, and United States	ART-naive, age ≥18 years, pVL >5 000 copies/ml	96 weeks	A:TDF + FTC + EFV, N = 284; B: TDF + FTC + RAL, N = 282
[31] Markowitz 2007, [32] Markowitz 2009, Protocol 004 part II, RCT, double-blinded, 29 centers in United States, Canada, Latin America, Thailand, and Australia	ART-naive (less than 7 days of ART was permitted), 30 patients received 10 days of RAL in monotherapy as a first part of the study**), age ≥18 years, pVL ≥5 000 copies/ml	96 weeks	A: TDF + 3TC + EFV, N = 39; B: TDF + 3TC + RAL (100 mg bid N = 41, 200 mg bid N = 40, 400 mg bid N = 41, 600 mg bid N = 40)
[33] Sax 2012, [34] Zolopa 2013, GS-US-236-0102, RCT, double-blinded, centers in North America	ART-naive, age ≥18 years, pVL ≥5 000 copies/ml	96 weeks	A: FTC + TDF + EFV, N = 354; B: FTC + TDF + EVG + COBI, N = 353
[35] van Lunzen 2012, [36] Stellbrink 2013, SPRING-1, RCT, open-label (only dose but not drug allocation was masked), 34 sites in France, Germany, Italy, Russia, Spain, and the United States	ART-naive (up to 10 days of ART was permitted), age ≥18 years, pVL>1 000 copies/ml	96 weeks	A: 2 NRTI + EFV, N = 50; B: 2 NRTI + DTG (10 mg N = 53, 25 mg N = 51, 50 mg N = 51); (2 NRTI included: TDF + FTC or ABC + 3TC)
[37] Walmsley 2013, SINGLE, RCT, double-blind, centers in North America, Europe, and Australia	ART-naive, age ≥18 years, pVL ≥1 000 copies/ml	48 weeks	A: TDF + FTC + EFV, N = 419; B: ABC + 3TC + DTG, N = 414
background regimen (2 or 3 NRTIs) + efavirenz vs background regimen + bPI			
[38] Albini 2012, RCT, open-label, 4 centers in Italy	ART-naive, age ≥18 years	48 weeks	A: TDF + FTC + EFV, N = 43; B: TDF + FTC + ATV/r, N = 48
[39] Bartlett 2006, CLASS, RCT, open-label, centers in United States	ART-naive (less than 2 weeks of prior ART), pVL ≥5 000 copies/ml	96 weeks	A: ABC + 3TC + EFV, N = 97; B: ABC + 3TC + APV/r, N = 96
[40] Cameron 2008, M03-613, RCT, open-label, centers in Canada, United States and Spain	ART-naive, pVL >1 000 copies/ml	96 weeks	A: AZT + 3TC + EFV, N = 51; B: AZT + 3TC + LPV/r, N = 104
[41] Daar 2011, A5202, RCT, open-label (only NRTI treatment was blinded), 59 centers in the United States and Puerto Rico	ART-naive (up to 7 days of ART was allowed), age ≥16 years	median—138 weeks	A: 2 NRTI + EFV, N = 929; B: 2 NRTI + ATV/r, N = 928; 2 NRTI included: ABC + 3TC or TDF + FTC***
[42] Echeverría 2010, LAKE, RCT, open-label, 19 centers in Spain and Italy	ART-naive, age ≥18 years	48 weeks	A: ABC + 3TC + EFV, N = 63; B: ABC + 3TC + LPV/r, N = 63
[43] Honda 2011, RCT, open-label, 1 center in Japan	ART-naive, CD4+ T lymphocyte 100–300 cell/mm3, men only	96 weeks	A: 3TC + ABC + EFV, N = 36; B: 3TC + ABC + ATV/r, N = 35
[44] Josephson 2010, [45] Edén 2010, [46] Andersson 2013, NORTHIV, RCT, open-label, centers in Sweden and Norway	ART-naive, age ≥16 years	144 weeks	A: 2 NRTI + EFV, N = 78 [46]; B: 2 NRTI + ATV/r, N = 82 [46]; C: 2 NRTI + LPV/r, N = 83 [46]; 2 NRTI included: ABC + 3TC or TDF + FTC OR AZT + 3TC or others
[47] Kumar 2013, SUPPORT, RCT, open-label, centers in United States	ART-naive (≤14 days of treatment with any ART), pVL >5 000 copies/ml	96 weeks	A: ABC + 3TC + EFV, N = 50; B: ABC + 3TC + FPV/r, N = 51
[47] Mallolas 2008, TRIZEFAL, RCT, open-label, 18 centers in Spain	ART-naive, pVL >10 000 copies/ml	72 weeks	A: ABC + 3TC + AZT + EFV, N = 109 (N = 104 were analyzed); B: ABC + 3TC + AZT + LPV/r, N = 111 (N = 105 were analyzed)
[49] Miró 2010, ADVANZ, RCT, open-label, 6 centers in Spain	ART-naive, age ≥18 years, CD4+ T lymphocyte <100 cells/μl	36 months	A: AZT + 3TC + EFV, N = 35; B: AZT + 3TC + IDV/r, N = 35
[50] Puls 2010, ALTAIR, RCT, open-label, 36 centers in Australia, Thailand, Argentina, France, Singapore and United Kingdom	ART-naive, age >18 years, pVL >2 000 copies/ml	48 weeks	A: TDF + FTC + EFV, N = 115; B: TDF + FTC + ATV/r, N = 107
[51] Ratsela 2010, PHISIDA II, RCT, open-label, 6 centers in the Republic of South Africa	ART-naive (less than 7 days of ART was allowed), age ≥14 years, CD4+ T lymphocyte <200 cell counts	median 24.7 months	A: 2 NRTI + EFV, N = 888; B: 2 NRTI + LPV/r, N = 883; 2 NRTI included: AZT + ddI or d4T + 3TC
[52] Riddler 2008, A5142, RCT, open-label, centers in United States, centre in Dublin and Durban	ART-naive, age ≥13 years, pVL ≥2 000 copies/ml	96 weeks	A: 2 NRTI + EFV, N = 250; B: 2 NRTI + LPV/r, N = 253; (2 NRTI included: 3TC and: AZT or d4T or TDF)
[53] Sierra-Madero 2010, RCT, open-label, 10 centers in Mexico	ART-naive, age ≥18 years, pVL ≥1 000 copies/ml	48 weeks	A: AZT + 3TC + EFV, N = 95; B: AZT + 3TC + LPV/r, N = 94
[54] Torti 2005,[55] Torti 2008, substudy of SISTHER, RCT, open-label, 1 center in Italy	ART-naive, pVL ≥1 000 copies/ml	52 weeks	A: TDF + 3TC + EFV, N = 37 [55], N = 10 [54]; B: AZT + 3TC + LPV/r, N = 27[55], N = 9 [54]; C: TDF + ddI + EFV#, N = 11 [54]
background regimen (2 NRTIs) + efavirenz vs background regimen + CCR5			
[56] Copper 2010, [57] Sierra-Madero 2010, MERIT, RCT, double-blinded, double-dummy, centers in North and South America, Europe, South Africa, and Australia	ART-naive, age ≥16 years, pVL ≥2 000 copies/ml	96 weeks##	A: AZT + 3TC + EFV, N = 361; B: AZT + 3TC + MVC, N-360
[58] Currier 2008, ASCENT, RCT, partially double-blinded, 33 centers in United States, 4 in Canada and 24 in European Union	ART-naive, age ≥13 years, pVL ≥10 000 copies/ml,	96 weeks###	A: 3TC + AZT + EFV, N = 29; B: 3TC + AZT + APL (600 mg N = 58, 800 mg N = 58)
[59] Landovitz 2008, RCT, double-blinded (centers not described)	ART-naive (less than 2 weeks of ART was allowed, patients in VCV arm received VCV for 14 days in monotherapy), age ≥18 years, pVL ≥5 000 copies/ml	48 weeks	A: AZT + 3TC + EFV, N = 24; B: AZT + 3TC + VCV, N = 68 (all doses combined)

3TC—lamivudine, ABC—abacavir, APL—aplaviroc, APV—amprenavir, ART—antiretroviral therapy, ATV—atazanavir, AZT—zidovudine, bid—twice a day, bPI—ritonavir-boosted protease inhibitor, CCR5—CC chemokine receptor type 5, COBI—cobicistat, d4T - stavudine, ddC—zalcitabine, ddI—didanosine, DTG—dolutegravir, EFV—efavirenz, ETV—etravirine, EVG—elvitegravir, FPV—fosamprenavir, FTC—emtricitabine, IDV—indinavir, MVC—maraviroc, LPV—lopinavir, LRV—lersivirine, NFV—nelfinavir, NNRTI—non-nucleoside reverse transcriptase inhibitor, NRTI—nucleoside reverse transcriptase inhibitor, NVP—nevirapine, PI—protease inhibitor, r—ritonavir, RAL—raltegravir, RCT—randomized controlled trial, RPV—rilpivirine, SQV—saquinavir, TDF—tenofovir, pVL—plasma HIV RNA, VCV—vicriviroc.

* interventions included in meta-analysis only.

**raltegravir monotherapy did not influenced the efficacy results, so both groups (pretreated and not pretreated with raltegravir were combined).

***results for groups assigned EFV + different NRTIs and separately ATV/r + different NRTIs were combined.

#enrolment in the TDF + ddI + EFV arm was stopped as soon as the high rate of virological failure was recognized.

##once-daily MVC arm was discontinued prematurely and not analyzed.

###stopped prematurely due to unexpected hepatotoxicity.

The characteristics of the included studies are presented in Table 3. Eleven studies were double-blind. Only four of the included studies provided information about allocation concealment. Most of the trials reported data about patient withdrawals and drop-outs from the study. Jadad scores ranged from 1 to 4, mostly due to a lack of blinding and insufficient data about randomization methods used (Table 2).

### Effectiveness of adding efavirenz vs non-nucleoside reverse transcriptase inhibitor (NNRTI) to the background regimen

**Fig 2:**
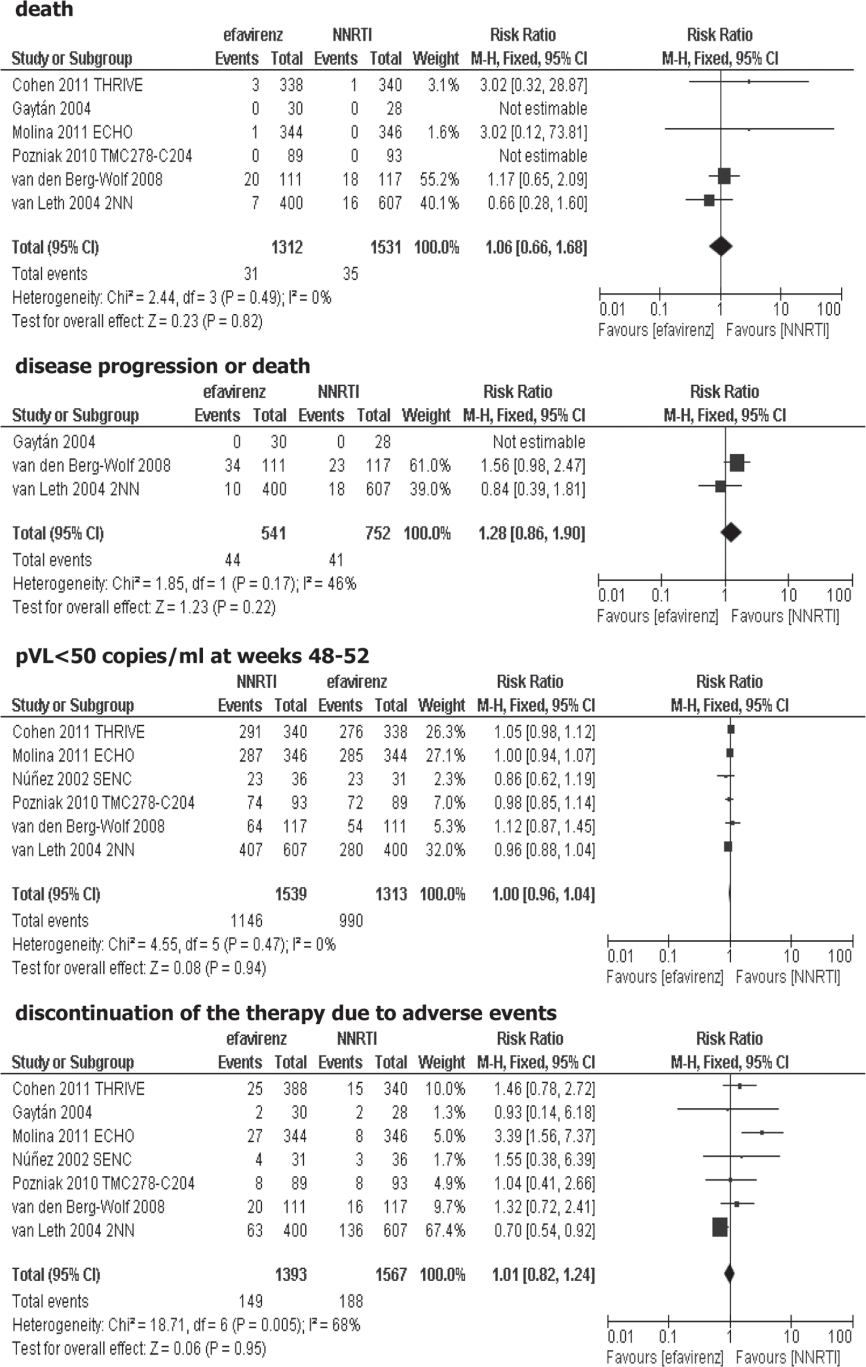
Forest plot of comparison: efavirenz vs other NNRTI added to the background regimen.

Ten studies were suitable for inclusion in the comparison of efavirenz vs. other NNRTI added to the background regimen: THRIVE [15], Gaytán [16], SENSE [17–18], ECHO [19], SENC [20], TMC278-C204 [21], NNRTI substudy [22] of the FIRST trial [23], 2NN [24], A5271015 [25], TSHEPO [26]. Nevirapine was used as a comparator in five studies [16,20,22,24,26], rilpivirine in three studies [15,19,21], while one study accessed etravirine [17–18], and one study included lersivirine as a comparator [25]. Data from SENSE trial [17–18] were excluded from meta-analysis because etravirine, according to the current guidelines, is not recommended as an initial therapy in HIV-infected patients, due to the insufficient data in antiretroviral-naive patients [6]. We also did not include results of A5271015 [25] study, because the clinical development of lersivirine for the treatment of HIV infection was discontinued in February 2013. With regard to TSHEPO [26] study, it was not possible to meta-analyze the outcomes due to their inconsistency with other trials. In FIRST substudy [22] and TMC278-C204 study [21], previous limited exposure to antiretroviral therapy was permitted (Table 3). In nevirapine studies, three of them evaluated patients during the course of 48 weeks while in two trials the follow-up period lasted 3 to 5 years (median). In all three studies, where rilpivirine was given to HIV-infected patients the follow-up period lasted 96 weeks. Studies were heterogeneous regarding baseline plasma HIV RNA level (>500 to >55 000 copies/ml). In two trials (Gaytán [16] and FIRST substudy [22]) it was unclear whether the efficacy results were obtained in ITT (intention-to-treat) population. Data for nevirapine given at different doses in two arms in the 2NN study [24] were aggregated. TMC278-C204 study [21] assessed rilpivirine at three doses: 25, 75 and 150 mg, however, data concerning only 25 mg dose were used in the meta-analysis, according to product characteristics [27]. No statistically significant differences between efavirenz and other NNRTI were observed when the primary efficacy endpoint—death was analyzed (RR = 1.06; 95% CI: 0.66–1.68; p>0.05), or composite outcome—disease progression or death was evaluated (RR = 1.28; 95% CI: 0.86–1.90; p>0.05). There were no statistically significant differences between analyzed groups in the proportion of patients with virological response at weeks 48–52 (plasma viral loads below 50 copies/ml: RR = 1.00; 95% CI: 0.96–1.04; p>0.05). Results of meta-analysis showed that the risk of discontinuation of assigned treatment due to intolerance was comparable in both arms, without statistically significant differences (RR = 1.01; 95% CI: 0.82–1.24; p>0.05), Fig 2. It should be noticed that although data from SENSE trial [17–18] were excluded from the above comparisons, we also tested whether inclusion of the data from this trial can change the cumulative effect. We found similar results on efficacy and safety based on cumulative data, regardless of the inclusion results from SENSE trial (data not shown).

### Effectiveness of adding efavirenz vs integrase strand transfer inhibitor (InSTI) added to the background regimen

**Fig 3:**
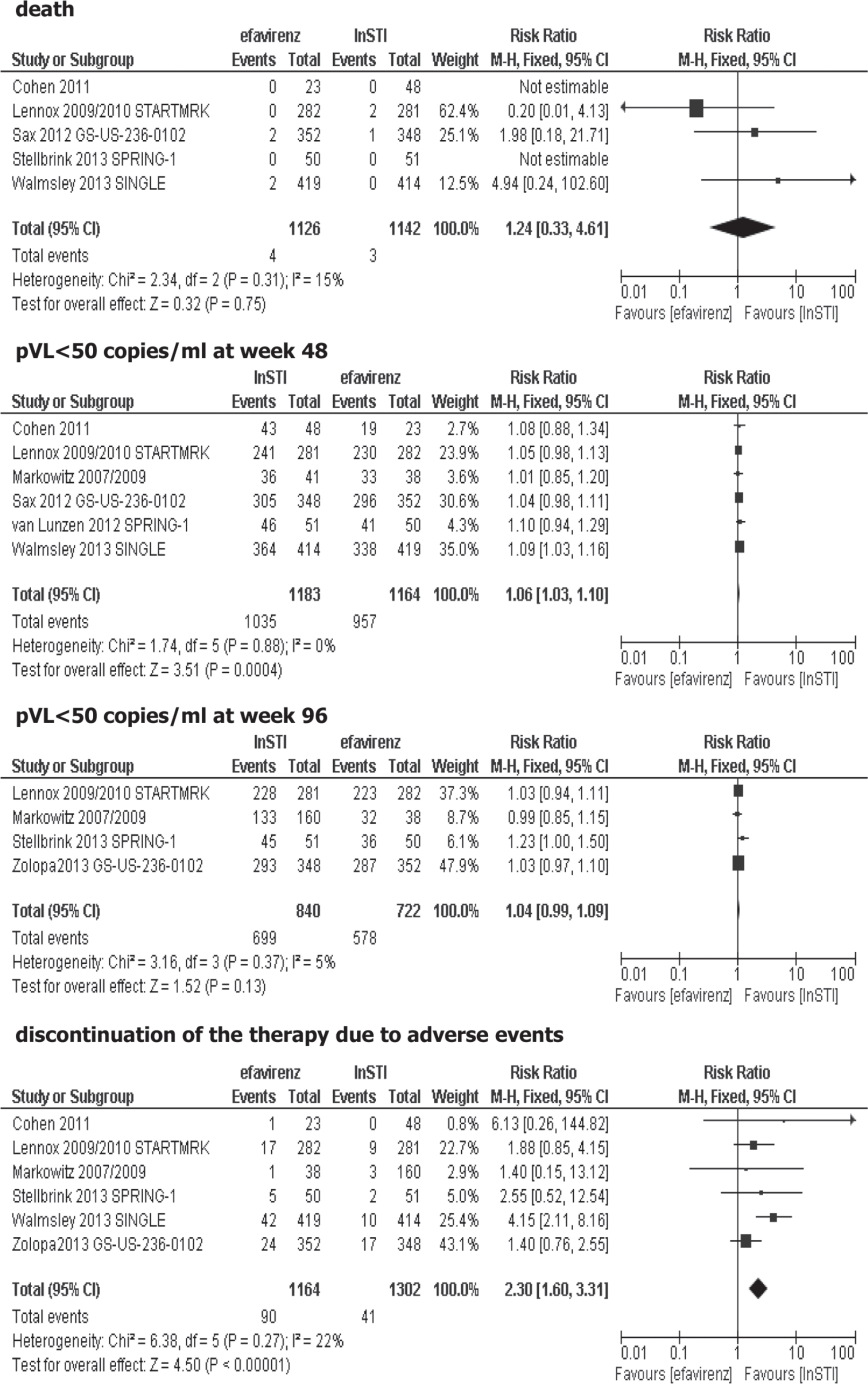
Forest plot of comparison: efavirenz vs InSTI added to the background regimen.

Six trials were included in the comparison between efavirenz and integrase strand transfer inhibitors added to the background regimen in the treatment of HIV-infected antiretroviral-naive patients: GS-236-014 [28], STARTMRK [29–30], Protocol 004 part II [31–32], GS-US-236-0102 [33–34], SPRING-1 [35–36] and SINGLE [37]. In the two trials, raltegravir was used as an InSTI [29–32], in the next two trials, elvitegravir in combination with cobicistat (a potent CYP 3A inhibitor that increases concentration of elvitegravir, and has no antiviral activity itself) were applied [28], [33–34], two studies assessed dolutegravir as an InSTI [35–37]. Results of Protocol 004 part II, STARTMRK, GS-US-236-0102 and SPRING-1 were reported separately for 48-week and 96-week follow-up period. Previous limited exposure to HAART was permitted in two trials: Protocol 004 part II [31–32], and SPRING-1 [35–36]. Four trials provided 96-week results: Protocol 004 part II, STARTMRK, GS-US-236-0102 and SPRING-1, while two studies comprised data only for 48 weeks of treatment: GS-236-014, and SINGLE. Most studies included patients with plasma viral load baseline of more than 5 000 copies/ml (only SPRING-1 [35–36] and SINGLE [37] study included patients with pVL>1 000 copies/ml). Protocol 004 part II [31–32] study assessed four different doses of raltegravir, however while only 400 mg of raltegravir twice a day is consistent with product characteristics, data for this dosage was included in the meta-analysis. After 48-weeks of treatment in Protocol 004 part II, raltegravir arms were combined and all group of patients were given 400 mg twice a day of raltegravir, therefore, data for 96 weeks were shown for all patients initially assigned raltegravir irrespective of the initial dose. Data for discontinuation of treatment due to clinical and laboratory adverse events from Protocol 004 part II study were combined [32]. SPRING-1 trial assessed three different doses of dolutegravir, however, since the study findings showed that 50 mg dolutegravir once daily is the most appropriate dose, data only for this dose were included in meta-analysis. Results of the present meta-analysis showed no statistically significant differences between efavirenz vs. integrase strand transfer inhibitor added to the background regimen for the following outcomes: death (RR = 1.24; 95% CI: 0.33–4.61; p>0.05) and proportion of patients with pVL<50 copies/ml at week 96 (RR = 1.04; 95% CI: 0.99–1.09; p>0.05). Statistically significant more patients treated with integrase strand transfer inhibitor achieved plasma viral load <50 copies/ml at week 48 (RR = 1.06; 95% CI: 1.03–1.10; p<0.05), while in terms of toxicity higher risk of discontinuation of therapy due to adverse events was reported when an efavirenz-based regimens were used (RR = 2.30; 95% CI: 1.60–3.31; p<0.05), Fig 3.

### Effectiveness of adding efavirenz vs ritonavir-boosted protease inhibitor (bPI) to the background regimen

**Fig 4:**
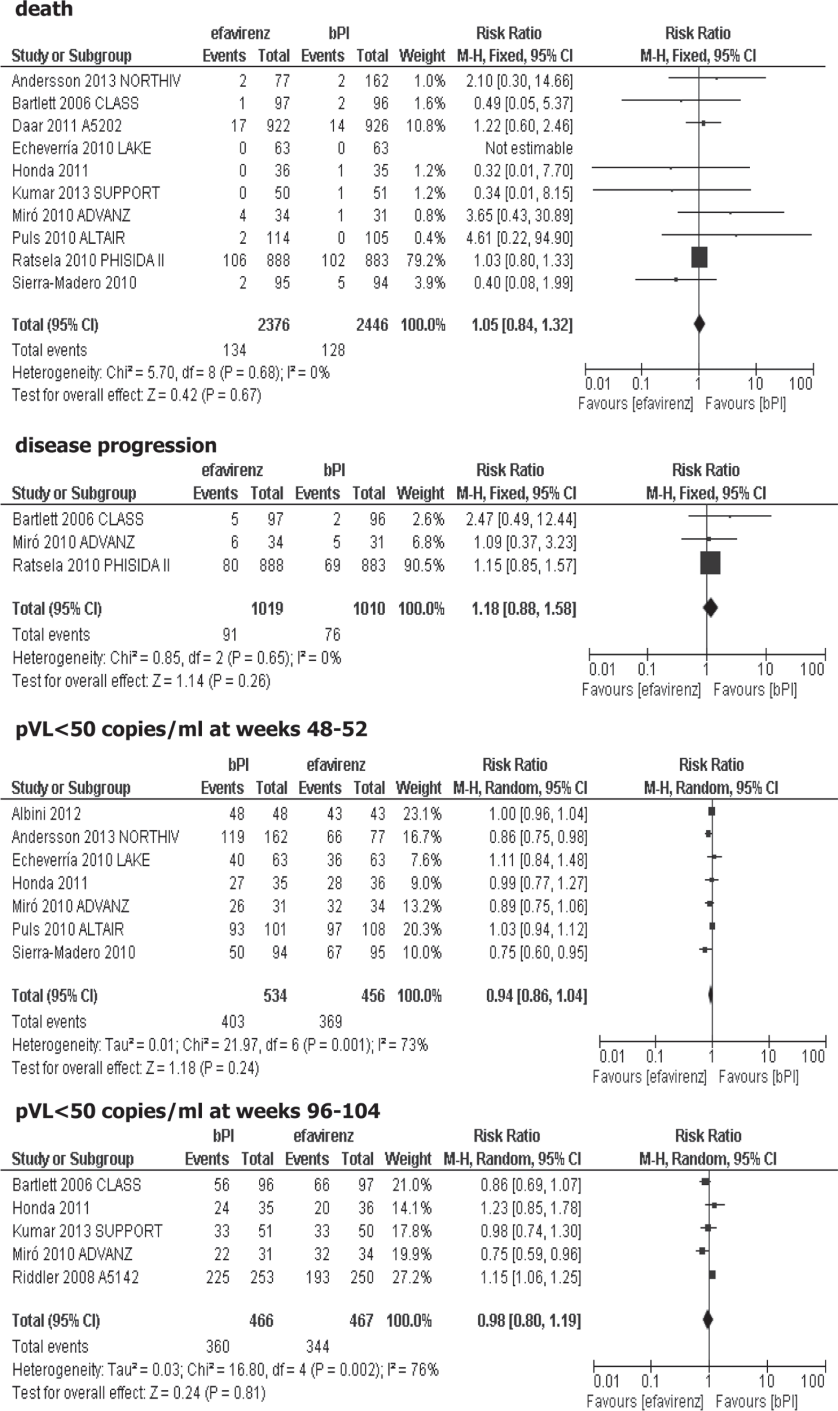
Forest plot of comparison: efavirenz vs ritonavir-boosted PI (bPI) added to the background regimen-efficacy data.

Fifteen studies were included in the comparison of efavirenz vs ritonavir-boosted protease inhibitor (bPI) added to the background regimen: Albini 2012 [38], CLASS [39], M03-613 [40], A5202 [41], LAKE [42], Honda 2011 [43], NORTHIV [44–46], SUPPORT [47], TRIZEFAL [48], ADVANZ [49], ALTAIR [50], PHISIDA II [51], A5142 [52], Sierra-Madero 2010 [53], substudy of SISTHER [54–55]. Lopinavir and atazanavir were used as protease inhibitors in eight and five trials, respectively, while amprenavir, indinavir and fosamprenavir were used in single studies, all of them were ritonavir-boosted. Six trials lasted 48–52 weeks, but the follow-ups of the remaining studies were longer (up to 36 months). Four studies additionally included patients with a limited previous exposure to HAART therapy (PHISIDA II [50], A5202 [41], CLASS [39], and SUPPORT [46]). Studies were heterogeneous in regard to the baseline plasma HIV RNA level (>1000 to >10 000 copies/ml), although, in many studies baseline plasma viral load was not a criterion of patient’s inclusion. In Albini 2012 study, the changes of GFR (glomerular filtration rate) during therapy were the primary endpoint [38]. With regard to TRIZEFAL [48] study, it was not possible to meta-analyze the efficacy outcomes due to their inconsistency with other trials, likewise in the substudy of SISTHER trial where plasma viral load was measured only at week 28 [54–55]. In the M03-613 study [40], after 24 weeks of combined therapy with 2 NRTI and lopinavir/ritonavir, the nucleoside reverse transcriptase inhibitor backbone was discontinued. That means 48-week results reflected a comparison of 2 NRTI+efavirenz and two-drug regimen of lopinavir/ritonavir without NRTI background and therefore, data from M03-613 trial [40] were not sufficiently homogeneous for inclusion in the current meta-analysis. The A5202 study did not provide information about the total number of patients in separate groups in whom measurement of pVL<50 copies/ml was performed at week 48 and 96, thus inclusion of this data in the current meta-analysis was not possible [41]. In NORTHIV trial, the results of the study were combined from both PI-based regimens [44–46]. No statistically significant differences between efavirenz and PI-based regimen were observed when the primary efficacy endpoints were analyzed: death (RR = 1.05; 95% CI: 0.84–1.32; p>0.05) and disease progression defined in three studies as an occurrence of AIDS-defining events (RR = 1.18; 95% CI: 0.88–1.58; p>0.05). There were also no statistically significant differences between the analyzed groups (efavirenz vs. ritonavir-boosted PI, both added to the background therapy) in the proportion of patients with plasma viral loads below 50 copies/ml at weeks 48–52 (RR = 0.94; 95% CI: 0.86–1.04; p>0.05) and at weeks 96–104 (RR = 0.98; 95% CI: 0.80–1.19; p>0.05), Fig 4.

**Fig 5:**
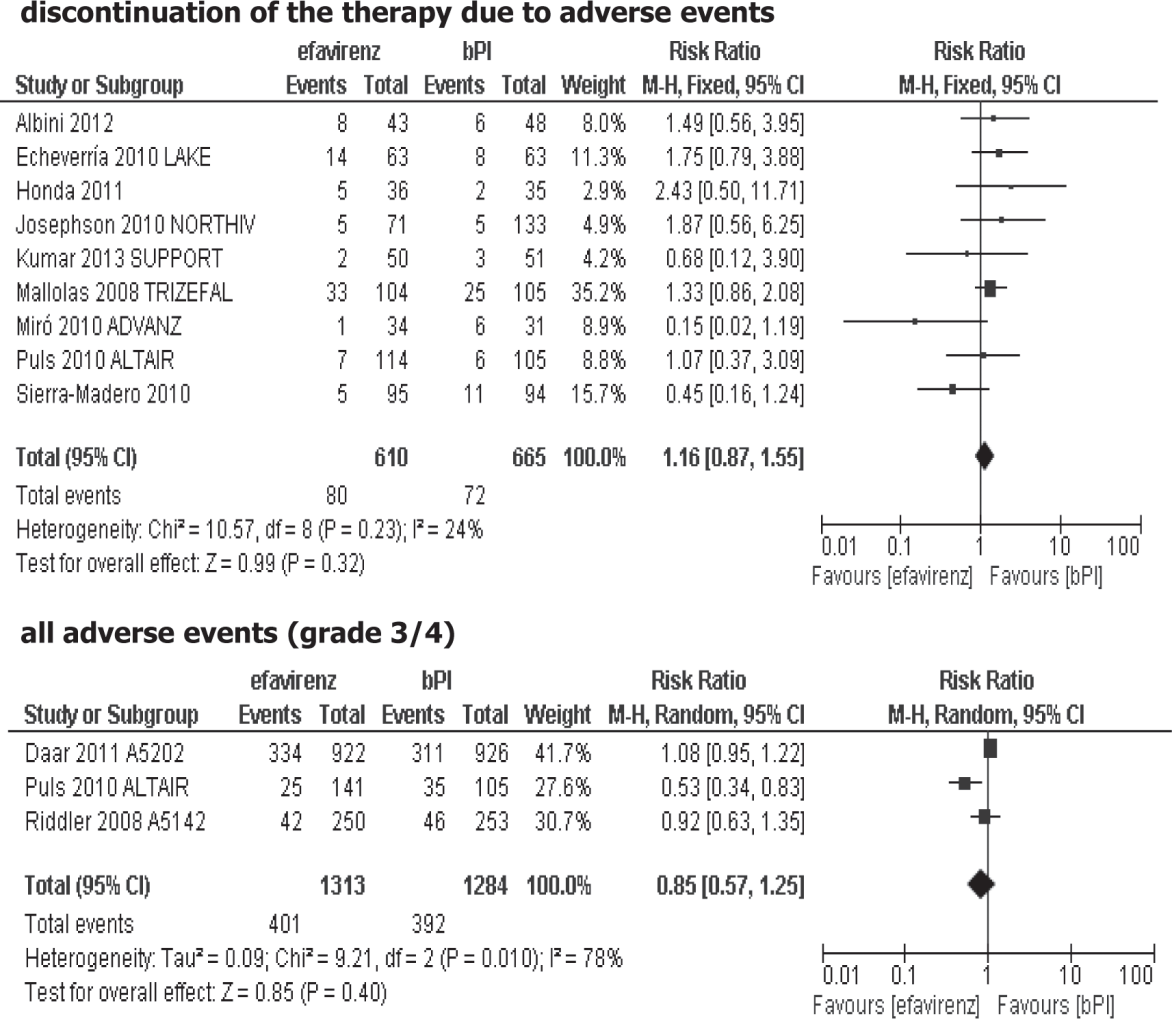
Forest plot of comparison: efavirenz vs ritonavir-boosted PI (bPI) added to the background regimen-safety data.

Results of meta-analysis showed that the risk of discontinuation of treatment due to its intolerance was comparable for both arms, without statistically significant differences (RR = 1.16; 95% CI: 0.87–1.55; p>0.05), and the risk of grade 3/4 adverse events was similar in both groups (RR = 0.85; 95% CI: 0.57–1.25; p>0.05), Fig 5.

### Effectiveness of adding efavirenz vs CC chemokine receptor type 5 (CCR5) antagonist added to the background regimen

Three trials were included in the comparison of efavirenz vs. CCR5 antagonists, both added to the background regimen in the treatment of HIV-infected antiretroviral-naive patients: MERIT [56–57], ASCENT [58], Landovitz 2008 [59]. Three different CCR5 antagonists were assessed in the above studies: maraviroc [56–57], aplaviroc [58] and vicriviroc [59]. Since clinical studies investigating aplaviroc were discontinued due to cases of hepatoxicity identified during phase II and III studies (including ASCENT study) [60] and currently this agent is not authorized for marketing in the United States and the European Union, results of ASCENT study were excluded from the present analysis. Unfortunately, it was not possible to meta-analyze the results of MERIT [56–57] and Landovitz 2008 [59] trials due to inconsistent efficacy and safety outcomes measured in the above-mentioned studies. It should be noted that results of the Landovitz 2008 study [59] showed higher rates of virologic failure in vicriviroc groups (25 mg and 50 mg once a day) compared with efavirenz group, suggesting that higher doses of vicriviroc should be investigated in the future clinical trials. Results from MERIT study were described in two references where data for 48-week and 96-week follow-up periods were reported [56–57]. Results for 96-week follow-up were assessed post-hoc in population of 614 patients with CCR5-tropic (R5) HIV-1 that was confirmed with greater sensitivity assay that during randomization [57]. Results of the present analysis (based on MERIT study results, data not shown) revealed no statistically significant differences between efavirenz and maraviroc added to the 2 NRTI for the clinical outcomes: death (RR = 1.00; 95% CI: 0.06–15.88; p>0.05) and disease progression defined as an occurrence of C category events indicating a development of AIDS (RR = 1.99; 95% CI: 0.76–5.26; p>0.05) at week 48. Likewise, risk of death in the two treatment arms did not differ statistically for 96-week follow-up period (RR = 1.50; 95% CI: 0.25–8.90; p>0.05). No statistically significant differences were observed between efavirenz and maraviroc in regard to virological outcomes: plasma viral load below 50 copies/ml at week 48 (RR = 0.94; 95% CI: 0.85–1.04; p>0.05) and at week 96 (RR = 0.94; 95% CI: 0.83–1.07; p>0.05). Comparison of efavirenz and CCR5 antagonist in terms of toxicity showed no statistically significant differences between the analyzed regimens in the risk of grade 3/4 adverse events at week 48 (RR = 1.23; 95% CI: 0.94–1.61; p>0.05) and at week 96 (RR = 1.16; 95% CI: 0.91–1.47; p>0.05), however, a significantly higher risk of discontinuation of therapy due to adverse events was observed during treatment with efavirenz-based regimen (RR = 3.26; 95% CI: 1.86–5.70; p<0.05), which was confirmed for both 48 and 96-week follow-up periods.

## Discussion

The choice of the first-line therapy (initial therapy) is determined by various considerations, which include severity of infection, drug tolerability, presence of drug-resistant mutations in non-treated populations, pregnancy or availability of drugs. The cost of therapy is also an important factor, especially in low-resource countries [61]. Difficulties in finding the optimal treatment option for HIV-infected patients which would provide optimal efficacy, durability of antiretroviral activity, tolerability and convenient dosing schedule are reflected by numerous antiretroviral regimens evaluated in clinical trials. Among the initial regimens, the most preferred by clinical experts is a backbone combination of two NRTIs—tenofovir disoproxil fumarate and emtricitabine plus an active drug from one of the following classes: NNRTI (efavirenz), PI (ritonavir-boosted atazanavir, ritonavir-boosted darunavir) or InSTI (raltegravir, dolutegravir) [6–8]. The recommended alternative regimens consist of active drugs which represent the following classes: NNRTI (rilpivirine and nevirapine), PI (fosamprenavir/ritonavir, lopinavir/ritonavir), InSTI (elvitegravir/cobicistat) and CCR5 antagonist (maraviroc) [6–8].

Efavirenz is one of the most commonly used antiretroviral drugs and is one of the preferred treatment options for HIV-infected antiretroviral-naive (ART-naive) patients [62]. To our knowledge this is the first such a broad systematic review containing meta-analysis comparing efavirenz-based therapy with other currently recommended antiretroviral regimens used to treat antiretroviral-naive HIV-infected adult patients. Our report is also the most recent systematic review regarding efavirenz-based regimens evaluated in randomized controlled trials conducted in population of HIV-infected patients previously untreated with antiretroviral therapy.

According to the current guidelines for the use of antiretroviral agents during the initial therapy of HIV-infected patients, comparisons were made between efavirenz and drugs from 4 different classes: NNRTI (other than efavirenz), ritonavir-boosted PI (bPI), InSTI and CCR5, all of them added to the backbone regimen. Our meta-analysis demonstrated that efficacy of all regimens based on the above-mentioned drugs were comparable, the analysis did not show any differences in terms of clinical endpoints (death or disease progression usually defined as an occurrence of AIDS-defining events). In terms of virological response (decline in plasma viral load to the predefined levels at week 48) only comparison of efavirenz vs. InSTI showed a statistically significant difference favoring integrase strand transfer inhibitor while in other analysis differences were not statistically significant. The results of the individual studies included in our comparison of efavirenz vs. integrase strand transfer inhibitors showed a trend toward a better effect of InSTI on virological response at week 48. Above effect in the individual studies was not statistically significant probably due to the small number of patients (studies: GS-236-014 [28], SPRING-1 [35–36], Protocol 004 part II [31–32]) while results of SINGLE [37] trial that recruited more than 800 patients showed a statistically significant difference favoring InSTI in comparison with efavirenz. Integrase strand transfer inhibitors are a promising group of antiretroviral agents. Results of a recent FLAMINGO study showed that dolutegravir was superior to darunavir plus ritonavir, both with combination with NRTIs in treatment of HIV-infected ART-naive patients [63]. It is worth noting that, given the results of the latest research Department of Health and Human Services (DHHS) added dolutregravir in combination with selected NRTIs as one of the preferred options in ART-naive HIV-infected patients [6].

Our results are generally in agreement with meta-analysis performed by the other authors. Mbuagbaw et al. [64] found no differences in efficacy between efavirenz and nevirapine-based therapy (irrespective of the dosage of nevirapine) applied as an initial treatment for the antiretroviral-naive, HIV-infected patients. Efavirenz and nevirapine compared as a part of a three-drug combination were both equally effective in the suppression of HIV infection for such outcomes as: virological success (percentage of participants achieving undetectable plasma HIV RNA concentration over time), clinical progression of AIDS and mortality. Similar observations were reported by Siegfried et al. [61] who demonstrated equivalent efficacy of two NNRTIs (efavirenz and nevirapine-based regimens) for the treatment of HIV infection in antiretroviral treatment-naive adults [61]. We also confirmed results of our previous meta-analysis where no statistically significant differences were shown between nevirapine and efavirenz in terms of disease progression or death as well as virological response and safety profile in antiretroviral-naive HIV-infected patients [65]. However meta-analysis of Pillay et al. showed that efavirenz-treated, treatment-naive patients were more likely to achive virologic success than patients treated with nevirapine (RR = 1.04; 95% CI: 1.00–1.08) [66]. It should be mentioned, that about half of the trials included to the above-mentioned meta-analysis [66] involved patients co-infected with tuberculosis, and hence these studies were excluded from our meta-analysis.

In terms of virological response our results are in contrast with the direct meta-analysis of Chou et al. which showed that NNRTI-based regimens in ART-naive patients were better than PI-based regimens with regard to decrease of plasma HIV RNA levels below 50 copies/ml [67]. However, the probable explanation for the observed discrepancy between both meta-analyses is that different trials were included. Chou et al. combined all studies evaluating drugs that belong to a NNRTI or PI class, while our analysis was consistent with the current guidelines for the first-line treatment. For this reason, we excluded studies assessing regimens not recommended for the first-line treatment (for example delavirdine as an NNRTI or unboosted-PI). In addition, the meta-analysis of Chou et al. [67] was performed in 2006, since that time the results of about 15 new RCTs have been published, which we could include in the current meta-analysis. Therefore, some of the regimens analyzed in our meta-analysis were not included in the previous one (for example rilpivirine as NNRTI or ritonavir-boosted atazanavir). Restriction to regimens including the older generation of PIs (that may be less potent) in the previous meta-analysis, could be the explanation of the observed differences between NNRTI and PI-based regimens [67], not confirmed by our cumulative results.

It should be noticed that the current meta-analysis described in this report has several limitations. The most important is that we were not able to aggregate results from studies comparing efavirenz and CCR5 antagonist-based regimens, due to inconsistent efficacy and safety outcomes measured in the included studies. For this reason, comparison of efavirenz and CCR5 antagonists was based on data from only one study, in which maraviroc was evaluated. The trial that was excluded from our meta-analysis (due to non-homogeneous outcomes in both trials evaluating CCR5 inhibitors) assessed vicriviroc, an experimental CCR5 antagonist, that is still in the phase of clinical trials and has not yet been approved for the treatment of HIV-infected, antiretroviral-naive patients. In addition, we did not identify the trials comparing a ritonavir-boosted darunavir (recommended by current guidelines) with efavirenz-based regimen in the analyzed population.

Another limitation was related to heterogeneity of the included studies regarding background regimens, baseline characteristics of randomized patients (especially proportions of patients in various stages of the disease), duration of follow-up periods, baseline median HIV RNA level, as well as differences in the definitions of the analyzed endpoints. While relatively low baseline HIV RNA plasma level is considered to be a predictive factor of virologic success during antiretroviral therapy [6,20], it should be noticed that the baseline plasma viral load varied within studies included in the meta-analysis (for example: pVL in studies included in the comparison of efavirenz and other NNRTI ranged from >5 000 copies/ml [15,17–19,24] to 100 000 copies/ml [20]). However, it should be emphasized that relatively large number of trials included in the individual meta-analysis (especially comparing NNRTI and ritnovir-boosted PI), reduced the influence of potential confounders and made the obtained results reliable.

Additionally, the absence of subgroup analysis in dependence on background regimens, due to a limited number of trials suitable for inclusion into separate comparisons, is another limitation of the present meta-analysis. A previous systematic review demonstrated that efavirenz is the most effective when administrated on a tenofovir and emtricitabine backbone [68], that is consistent with the currently published treatment guidelines for antiretroviral-naive patients [6,8]. The above-mentioned results indicate that backbone regimen may affect the results obtained in separate comparisons (when background regimen consisted of 2 NRTI was different in both treatment arms). However, it should be mentioned, that in most included studies backbone regimen was the same in the compared groups. Moreover, in the most recently published trials (assessing rilpivirine or InSTI), the already established and recommended combination of tenofovir and emtricitabine was used as a backbone regimen.

In this systematic review, we did not analyze trials including patients with comorbid, clinically relevant illnesses. There were several reasons for that, mainly the existence of separate recommendations for these groups of patients. Firstly, clinical guidelines recommend efavirenz as an initial ART regimen for patients on rifampin-based treatment for tuberculosis (while other NNRTIs as nevirapine are not recommended) [7–8]. Moreover, co-administration of rifampin and PIs (with or without ritonavir boosting) is not recommended [6], that restricts treatment options in those group of patients mostly to efavirenz-based therapy. Secondly, a systematic review and meta-analysis of studies, in which HIV/HCV co-infected patients were included, showed that statistically less patients with HIV/HCV co-infection reached plasma viral load <50 copies/ml at week 48 than HIV mono-infected patients (68,2% vs 80,4% respectively) [69]. In addition, patients with concurrent illnesses are at higher risk of adverse events leading to therapy discontinuation, moreover, a larger number of pills they have to take probably decreases the adherence to assigned treatment. What is more, interactions between the administered drugs should be also taken into consideration. We decided that the above factors could increase the heterogeneity of the included studies and decrease reliability of cumulative results of the meta-analysis and therefore we excluded such studies from our review.

The overall toxicity profile of efavirenz-based and other assessed regimens was comparable. However, a significantly higher risk of the discontinuation of therapy due to adverse events was observed in efavirenz group when compared with integrase inhibitors and CCR5 antagonists. It should be noticed that comparison with CCR5 antagonists was based on data from only one study and the reliability of these results is limited (further studies may change these preliminary conclusions). We did not analyze particular adverse events observed during treatment with of efavirenz-based regimens, however, neuropsychiatric adverse events were the most common side effects associated with efavirenz, that can limit the use of this agent [62]. Four systematic reviews regarding neuropsychiatric adverse events associated with efavirenz treatment have been recently published [62,70–72]. A systematic review performed by Kenedi et al. [62] demonstrated high rates of neuropsychiatric side effects among efavirenz-treated patients; vivid dreams, insomnia and mood changes were reported in approximately 50% of patients initiating treatment with efavirenz [62]. In antiretroviral-naive patients receiving efavirenz-based therapy, significantly higher rates of grade 1–4 neurological and psychiatric adverse events were reported compared to other regimens based on protease inhibitors or other NNRTIs [70]. Unfortunately, due to a different classification system used to access the above side effects reported in particular studies, limited data were suitable for comparison [70]. It should be noticed that most of the efavirenz-associated neuropsychiatric adverse events were generally mild and transient in nature. Based on the findings from clinical trials, efavirenz should not be initiated in patients with a history of severe psychiatric disorders, active mental illnesses, depressive symptom or severe sleep disorders in spite of the convenience of once daily dosing [62,70]. Recently published meta-analysis showed that patients receiving efavirenz were more likely to experience severe central nervous system-related adverse events than nevirapine [71]. Clinical experts indicate the need for large randomized controlled trials to determine if the neuronal toxicity induced by efavirenz results in clinically significant neurological impairment [72]. Despite the increased risk of neuropsychiatric adverse events in some of predisposed patients, efavirenz seems to have an acceptable safety profile. What is important, a meta-analysis of Gazzard et al. 2010 demonstrated no statistically significant differences between efavirenz and other first-line antiretroviral agents in regards to the incidence of any other side effects even such as severe or life-threatening adverse events [69].

Some authors suggest increased risk of suicide during efavirenz treatment. Recent meta-analysis of four randomized controlled trials showed an increased rate of suicidality events (including also suicidal ideation) in patients treated with efavirenz-containing antiretroviral regimen compared to other regimens. However while only completed/attempted suicides where assessed only a trend towards higher rates of above events in efavirenz group was reported [73]. Mentioned results were not confirmed in observational study where no higher death rates from suicide amongst patients treated with efavirenz than other regimens were reported [74]. What is more no evident association between efavirenz use and suicidality was reported when data from Food and Drug Administration Adverse Event Reporting System (FAERS) database were analyzed [75].

Results of the current meta-analysis strengthen the current clinical guidelines where active drugs from four different classes of antiretroviral agents are recommended. However, it should be noticed that some regimens can be recommended for a specific population of patients. For example rilpivirine is now recommended in patients with pretreatment HIV RNA ≤100,000 copies/mL, while the combination of elvitegravir/cobicistat/tenofovir/emtricitabine is recommended for patients with pre-treatment creatinine clearance >70 mL/min [6].

In conclusion, the results of the present meta-analysis support the current clinical guidelines for antiretroviral-naive, HIV-infected patients. Efavirenz-based therapy should be considered as one of the most preferred treatment options in ART-naive patients, however it should be prescribed with caution in patients with underlying psychiatric conditions. Results of recent studies suggests good efficacy and beneficial safety profile of drugs from new classes of antiretroviral agents (integrase inhibitors, CCR5 anatagonists) compared with other initial regimens used nowadays in clinical practice for the treatment of HIV-infected patients, however more data from further, reliable RCTs are needed to confirm above results.

## Supporting Information

S1 PRISMA Checklist (DOC)
